# Postponed Dental Appointments Due to Costs Are Associated with Increased Loneliness—Evidence from the Survey of Health, Ageing and Retirement in Europe

**DOI:** 10.3390/ijerph18010336

**Published:** 2021-01-05

**Authors:** Carolin Walther, Ghazal Aarabi, Richelle Valdez, Kristin Spinler, Guido Heydecke, Elzbieta Buczak-Stec, Hans-Helmut König, André Hajek

**Affiliations:** 1 Center for Dental and Oral Medicine, Department of Prosthetic Dentistry, University Medical Center Hamburg-Eppendorf, 20246 Hamburg, Germany; g.aarabi@uke.de (G.A.); r.valdez@uke.de (R.V.); kr.spinler@uke.de (K.S.); g.heydecke@uke.de (G.H.); 2 Center Psychosocial Medicine, Institute of Medical Sociology, University Medical Center Hamburg-Eppendorf, 20246 Hamburg, Germany; 3 Hamburg Center for Health Economics, Department of Health Economics and Health Services Research, University Medical Center Hamburg-Eppendorf, 20246 Hamburg, Germany; e.buczak-stec@uke.de (E.B.-S.); h.koenig@uke.de (H.-H.K.); a.hajek@uke.de (A.H.)

**Keywords:** dental appointments, dental care, dental health services, health services accessibility, dental avoidance, loneliness

## Abstract

As is already well known, demographic changes will presumably lead to a rising number of old aged individuals and loneliness is a tremendous concern in aging populations. Poor health can be a potential consequence of loneliness, as well as a determining factor. Thus, the objective of the current study was to determine whether postponed dental appointments due to costs affect loneliness longitudinally. Focusing on Germany, data from two waves (waves 5 and 6) of the “Survey of Health Ageing, and Retirement in Europe” (SHARE) were analyzed (*n* = 7703). The three-item loneliness scale (modified version of the revised UCLA Loneliness scale) was used to quantify loneliness. The presence of postponed dental appointments due to costs in the past 12 months (no; yes) served as a key independent variable. Socioeconomic factors as well as health-related factors were adjusted in the longitudinal regression analysis. After adjusting for confounding variables, regression analyses revealed that loneliness increased with decreases in self-rated health among men. Among women, loneliness increased when self-rated health decreased, when fewer chronic diseases and postponed dental appointments due to costs were reported. Among older women, postponed dental appointments due to costs are associated with feelings of loneliness. The study results add evidence that proper dental care (i.e., regular and appropriate visits to the dentist) is vital not only to one’s oral health, but also plays a role in one’s physical and emotional health.

## 1. Introduction

People suffering from untreated oral diseases increased from 2.5 billion in 1990 to 3.5 billion in 2015 [1]. Despite rising awareness of the potential consequences of chronic diseases among patients and dentists, non-attendance still occurs [2]. Data from the 2004 National Health Interview Survey of the Belgian population showed that almost one-half of the participants (49.7%) did not visit a dentist in the last 12 months. A low level of education, household income, dentate status and male gender were reasons for non-attendance at dental appointments [3]. Some of the reasons for this are based on individual choices, but some are based on adjustable political factors, such as sickness and rehabilitation benefits, maternity and child benefits, unemployment benefits, housing policies, social inclusion and care facilities [4]. Many patients postpone their dental appointments because they are simply unable to pay the upcoming costs due to the personal financial burden. In Germany, statutory health insured patients usually face significant private co-payments for removable or fixed dentures. However, there are a few exceptions: the treatment of acute tooth pain is usually free of charge. Additionally, one must keep in mind: Germany is still one of the few countries that even provides a social health insurance system for its citizens [5]. This phenomenon can be observed in other countries too. A recent study analyzed six Canadian surveys and found that the insurance coverage of middle-income citizens in Canada was generally low with a simultaneously high increase in perceived cost barriers to dental care [6]. In addition, a study conducted in Australia, which operates primarily with private dental systems, found that over two-thirds of people (*n* = 1083) avoid the dentist or visit less often than they felt they needed to. Again, and in agreement with others [7], the main reason for dental avoidance was costs for treatment [8]. Untreated or chronic oral diseases can affect patients’ general well-being: oral diseases are known to interact with other systemic diseases. For example, chronic oral inflammation is suspected to modulate the pathogenesis of atherosclerosis [9]. A retrospective cohort study, using the Taiwanese National Health Insurance Research Database (*n* = 393.745), indicated an increased risk of atrial fibrillation or flutter in patients with periodontitis [10]. In fact, the connection between periodontitis and systemic diseases is now very well studied [9,11]. However, untreated oral conditions not only affect the physical health of a patient but also their mental health. Previous studies suggest that people with poor oral health have a significantly higher risk of feeling lonely than participants with good oral health [12] and that low demand for and postponement of dental services may be linked with loneliness. Until now, only very few studies have examined the association between non-attendance to dental appointments and social isolation [13,14]. Loneliness in older adults is a topic of great concern; this phenomenon is even being referred to as a global “loneliness epidemic” [15]. It has been suggested that more than one-third of older adults perceive a lack of meaningful relationships or social engagements [16,17], especially in individuals with comorbidities (e.g., heart disease, depression) [18]. Being lonely or socially isolated has various consequences, such as emotional and mental effects (e.g., stress and depression, perceived lower quality of life). Risk factors for loneliness in older adults include depression, living alone, marital status (i.e., widowhood), low socio-economic status (SES), being female, and poor functional health [19].There is an urgent need to improve our understanding of dental coverage and close the potential knowledge gap of treatment cost-related reasons for postponement of dental appointments and the association with loneliness. Understanding the consequences is important—not only for health care policy and for health care providers, but also for developing health programs that encourage people to use dental services to promote their oral health and overall well-being.

Therefore, the research question of this study is as follows: is the postponement of dental appointments due to costs associated with loneliness longitudinally in a sample of older adults in Germany, aged 50 years and older? The conducted analyses were additionally stratified by gender because previous research suggests that there are gender differences in coping strategies between women and men [20]. Based on the few studies already conducted about loneliness and postponement of dental care, we hypothesize that there will be a significant association between postponed dental appointments and feelings of loneliness.

## 2. Materials and Methods

### 2.1. Participants

The study population was selected from waves 5 (year 2013) and 6 (year 2015) of the Survey of Health Ageing, and Retirement in Europe (SHARE) [21] due to reasons of data availability. More precisely, loneliness was not quantified in previous waves of the SHARE study.

The SHARE study is a multidisciplinary longitudinal study of non-institutionalized individuals’ ≥50 years in Europe and Israel focusing on social and family networks, socio-economic status and health. In the selected households, all individuals (and their partners independent of their age) ≥50 years were interviewed. Exclusion criteria were as follows: incarceration, hospitalization, being out of the country during the whole survey period, inability to speak the country’s language(s) or moving to an unknown address.

More information on SHARE has been described in further detail by Börsch-Supan et al. [22]. Because of data availability, we narrowed our analyses to Germany and previous waves were excluded. The final sample was composed of 7703 observations.

### 2.2. Outcome Measure: Loneliness

The three-item loneliness scale was used to quantify loneliness [23] in the SHARE study. It is a modified version of the Revised UCLA Loneliness scale [24,25]. A three-point Likert-scale was used (1 = “often”, 2 = “some of the time”, 3 = “hardly ever or never”). A sum score was computed by summarizing the responses for each of the three items.

Thus, the scale ranges from three (not lonely) to nine (very lonely). The three items are: How often do you feel a lack companionship? How often do you feel left out? How often do you feel isolated from others? Previous research has shown that the UCLA loneliness scale has favorable psychometric characteristics [23].

### 2.3. Independent Variables

Postponed dental appointments due to costs were measured with the question: “In the last twelve months, to help you keep your living costs down, have you postponed visits to the dentist?” [no; yes]. We included age, marital status (married and living together with spouse; registered partnership; married, living separated from spouse; never married; divorced; widowed), as well as income as covariates. In addition, morbidity was measured based on a count score (hypertension or high blood pressure; high blood cholesterol; cerebral vascular disease or stroke; high blood sugar or diabetes; arthritis, including rheumatism or osteoarthritis; chronic lung disease; malignant tumor or cancer; stomach or peptic ulcer, duodenal ulcer; Parkinson’s disease; cataracts; femoral or hip fracture). Self-rated health (from 1 = excellent to 5 = poor) was also included in the regression analysis. The regression analysis was stratified by sex.

### 2.4. Statistical Analysis

Fixed effects (FE) regressions were used to analyze data, since it has been shown that adjusting for unobserved heterogeneity is a key aspect of research in the area of subjective well-being [26]. Based on rather weak assumptions, FE regressions provide consistent estimates, even if unobserved factors are correlated with explanatory variables [27]. FE regressions are based on differences within participants (for the current study: individuals) over time. Therefore, time-constant factors such as sex cannot be included as covariate (since they do not have within variation). However, these time-constant factors are implicitly controlled in FE regressions. For example, differences in self-rated health within individuals over time are used for regression analyses. The choice to use FE regression analysis was supported by a Sargan-Hansen test (which is equal to a Hausman test with cluster-robust standard errors; in our study, the Sargan-Hansen statistic was 41.6, *p* < 0.001). Furthermore, using normal-probability plots, the normality assumption of the residuals was checked—indicating that the residuals were roughly normally distributed.

Cluster-robust standard errors were used. Statistical significance level at *p* < 0.05 was accepted. Statistical analyses were performed using Stata 15.1 (Stata Corp., College Station, TX, USA).

## 3. Results

### 3.1. Sample Characteristics

Stratified by sex, sample characteristics are displayed in Table 1 (observations used in linear FE regressions). In women, mean age was 65.3 years (SD: 9.9 years), and in men it was 66.2 years (SD: 9.7 years). Mean loneliness score was 3.8 (SD: 1.3) in women and 3.7 (SD: 1.1) in men. Further details are depicted in Table 1.

### 3.2. Regression Analysis

Findings of linear FE regressions are displayed in Table 2. Beta-coefficients are displayed. We reported sex-stratified regressions since our findings may vary depending on sex. Regressions showed that loneliness increased with worsening self-rated health among men (β = 0.13, *p* < 0.001). Among women, loneliness increased with decreases in self-rated health (β = 0.17, *p* < 0.001), fewer chronic diseases (β = −0.09, *p* < 0.05) and postponed dental appointments for financial reasons (β = 0.31, *p* < 0.05). Further details are depicted in Table 2.

In the total sample (i.e., not stratified by sex), there was also a link between increased loneliness and postponed dental appointments for financial reasons (β = 0.05, *p* = 0.047).

## 4. Discussion

### 4.1. Main Findings

Based on longitudinal data from the SHARE study, the aim of this study was to determine whether postponed dental appointments due to costs are associated with loneliness longitudinally. The analyses of this sample’s characteristics showed that men had a higher household income, were older on average, had more chronic diseases, were more often in a relationship, and more often reported not postponing dental appointments due to cost in comparison to women. Average loneliness scores were higher in women in comparison to men. The un-stratified regression analysis (total sample, not stratified by gender) showed a significant association between postponed dental appointments due to costs and increases in loneliness scores. For the gender-stratified FE regressions, it was revealed that there is significant association between postponing dental appointments for financial reasons and higher loneliness scores in women, even after adjusting for confounding factors. The hypothesis was, thus, accepted. Loneliness was also observed in both genders to have significant associations with having worse self-rated health and having fewer chronic diseases.

### 4.2. Previous Research and Possible Explanations

As is already well known, demographic changes will presumably lead to a rising number of old aged individuals and loneliness is a tremendous concern in aging populations [12]. The consequences for people who feel lonely can range from a general decrease in well-being to sleep disorders, depression and many more symptoms [28,29]. Thus, if one’s mental health or illness can affect one’s general health, it can be assumed that this causality is bidirectional. In other words: poor health can be a potential consequence of loneliness, as well as a determining factor [12]. Supporting these results, a longitudinal study of aging Finns in Southern Finland showed that better self-rated health is associated not only with the absence of loneliness, but also with its decrease [30]. A recent study used data from the English Longitudinal Study of Ageing (ELSA) and demonstrated that older individuals with poor oral health had significantly higher risks of feeling lonely than participants with sufficient oral health both cross-sectionally and longitudinally. Thus, the potential pathway linking oral deficits with loneliness appears not to be from loneliness to deficits in health (and oral health), but rather the other way around [12]. However, a focus group of 85-year-old individuals in Gothenburg (N = 454) reported less frequent dental appointments when they reported feelings of loneliness [13]. Loneliness is often connected with financial worries and besides low self-initiative, this seemed to be a significant reason for postponement of dental appointments. Furthermore, another study employed data from the 2008 Health and Retirement Study, and assessed that perceived social isolation was related to a lower probability of seeking dental care [14]. Thus, a reverse causality, meaning loneliness affects postponing dental appointments due to costs, cannot be dismissed. Although loneliness will become an increasing problem in our society, the risk factor ‘oral health’ has so far been seldom discussed. Therefore, one can only speculate about a possible explanation for our findings: We already know that dental appearance is becoming equally important in both younger and older individuals [31]. However, if a patient postpones dental appointments for financial reasons, the necessary treatment of oral diseases can no longer take place, with the possible consequences of “drifting teeth”, “loose teeth”, “swollen gums”, “sore gums”, “receding gums” and “bad breath” [32]. Furthermore, older individuals with poor oral health also experience poor eating function, problems with speaking, communication and emotional challenges. It is not surprising that even one of these limitations or all limitations combined lead to lower self-esteem, reduced self-confidence [33] and possibly also loneliness. Within this structure of logic, another aspect needs to be considered. If older individuals cannot attend dental appointments due to costs, it might be speculated whether this experience triggers the thought that they are worse off compared to others who can easily afford dental treatment. These social comparisons and the corresponding feelings (e.g., embarrassment, feelings of shame) can reinforce the feeling of loneliness/isolation [34].

Our results provide further evidence for the potential role of postponing dental appointments for financial reasons and the potential association with loneliness.

### 4.3. Strengths and Limitations

Our study is the first to examine the link between postponed dental appointments for financial reasons and feelings of loneliness. A nationally representative longitudinal database was used in this study. Fixed effects (FE) regressions were used to analyze data, since adjusting for (unobserved) heterogeneity is a key aspect of research in the field of well-being. A validated tool was used to assess loneliness. It becomes clear that the current study design cannot address the issue of causal direction. Furthermore, a small sample selection and a small attrition bias have been reported for the SHARE study [35]. Moreover, future studies should include further covariates related to dental health.

## 5. Conclusions

Among older women, postponing dental appointments for financial reasons is (strongly) associated with loneliness. Preventing postponement of dental appointments due to costs may be beneficial for loneliness scores among older women. The results of this study add evidence that proper dental care (namely, regular and appropriate visits to the dentist) is vital not only to one’s oral health, but also plays a role in one’s physical and emotional health as well. Policies to improve the accessibility of dental care (e.g., subsidization of necessary dental care for older persons) seems necessary. The improvement of pathways of information to educate older adults about the importance of oral health not only in and of itself, but also its influence on one’s overall physical and emotional well-being could also be provided. Additionally, information about possible financial help for dental care through awareness campaigns may prove beneficial in this population. Ideas for future research include exploring the causal direction of the association with postponement of dental appointments or exploring gender differences in the determinants of postponement of dental appointments, which may shape more effective interventions in older adults.

## Figures and Tables

**Table 1:** Characteristics of observations included in fixed effects regression analysis stratified by sex (*n* = 7703; wave 5 and wave 6; Germany)**.**

Variables	Male (*n* = 3645)		Female (*n* = 4058)	
	Mean	SD	Mean	SD
Age in years	66.2	9.7	65.3	10.0
Household net income (per year) in Euro	33,530.9	41,940.3	29,858.4	39,536.9
Number of chronic diseases	1.3	1.3	1.1	1.2
Self-rated health (1 = “excellent” to 5 = “poor”)	3.2	1.0	3.2	1.0
Loneliness (Loneliness Scale)	3.7	1.1	3.8	1.3
	N	%	N	%
Marital status: married and living together with spouse; registered partnership	2660	73.0%	2635	64.9%
Postponed dental visits for financial reasons: No	3514	96.4%	3877	95.5%

**Table 2:** Determinants of loneliness stratified by sex. Results of linear fixed effects regressions (wave 5 and wave 6; Germany).

Independent Variables	Loneliness Men	Loneliness Women
Postponed dental visits for financial reasons (Reference category: Not having postponed dental visits for financial reasons)	0.04	0.31 *
	(0.17)	(0.14)
Age (in years)	−0.01	−0.01
	(0.01)	(0.02)
Marital status: married and living together with spouse or registered partnership (Reference category: others married (living separated from spouse; never married; divorced; widowed))	−0.33 +	−0.59 +
	(0.18)	(0.35)
Log household net income (in Euro)	−0.06	−0.01
	(0.04)	(0.05)
Self-Rated health (1 = “excellent” to 5 = “poor”)	0.13 ***	0.17 ***
	(0.04)	(0.05)
Number of chronic diseases (ranging from 0 to 11)	−0.01	−0.09 *
	(0.04)	(0.04)
Constant	4.56 ***	4.42 ***
	(1.10)	(1.19)
Observations	3645	4058
Number of Individuals	2417	2696
R²	0.014	0.026
Sigma_u (SD of residuals within groups u_i_)	0.999	1.156
Sigma_e (SD of residuals (overall term) e_i_)	0.754	0.880
Rho	0.637	0.633

Notes: Beta-coefficients were reported; cluster-robust standard errors in parentheses; *** *p* < 0.001, * *p* < 0.05, + *p* < 0.10.

## Data Availability

Restrictions apply to the availability of these data. Data was obtained from a third party and cannot be made publicly available. More specifically, data for the study came from the SHARE project and are available to all researchers for purely scientific purposes upon request on their website (http://www.share-project.org/). Contact information: care of Josette Janssen; Address: CentERdata, Tilburg University, P.O. Box 90153, 5000 LE Tilburg, The Netherland. Email: jjanssen@uvt.nl. Fax: +31-13-4662764).
